# Dietary zerumbone prevents against ultraviolet B-induced cataractogenesis in the mouse

**Published:** 2011-03-12

**Authors:** Bo-Yie Chen, David Pei-Cheng Lin, Kuo-Chen Su, Yi-Ling Chen, Chia-Yung Wu, Mei-Ching Teng, Yuan-Ting Tsai, Chi-Yun Sun, Soo-Ray Wang, Han-Hsin Chang

**Affiliations:** 1 School of Optometry, Chung Shan Medical University, Taichung, Taiwan, ROC; 2 School of Medical Laboratory and Biotechnology, Chung Shan Medical University, Taichung, Taiwan, ROC; 3 Department of Ophthalmology, Chung Shan Medical University Hospital, Taichung, Taiwan, ROC; 4 Department of Ophthalmology, Chang Gung Memorial Hospital, Kaohsiung, Taiwan, ROC; 5 Department of Internal Medicine, Chung Shan Medical University Hospital, Taichung, Taiwan, ROC; 6 School of Nutrition, Chung Shan Medical University, Taichung, Taiwan, ROC

## Abstract

**Purpose:**

To investigate the preventive effect of dietary zerumbone against UVB-induced cataractogenesis.

**Methods:**

A total of 50 six-week-old female ICR mice were split into five groups (each contained 10 mice) and exposed to UVB (0.72 J/cm^2^/daily) at noon for 7 days, except for the blank control group. The mice with UVB exposure were fed with zerumbone as a dietary supplement at 0, 1, 10, and 100 mg/kg of bodyweight, respectively, starting from one day before UVB exposure. On day 7, at 4 h after UVB exposure, all mice were subjected to cataract examination and lens opacity scoring, in correlation with levels of MDA (malondialdehyde), GSH (glutathione), GR (GSH reductase), GPx (glutathione peroxidase), and SOD (superoxide dismutase) in the lens.

**Results:**

Dietary zerumbone at 100 mg/kg after UVB exposure was effective in decreasing lens opacity scores (p<0.001) and to reduce MDA (p<0.001), while GSH and GR levels were significantly increased (both p<0.001) in the lens. SOD was also increased with dietary zerumbone at 100 mg/kg (p=0.115), whereas GPx (p=0.171) levels were lower as compared with those without zerumbone after UVB exposure.

**Conclusions:**

These results suggest that zerumbone may protect against UVB-induced cataractogensis through reducing lipid peroxides and enhancing the endogenous antioxidant GSH level and GR activity.

## Introduction

Cataract formation is one of the leading causes of human blindness worldwide. Most cataracts are not congenital and ultraviolet radiation from sunlight plays a paramount role during cataractogensis, as epidemiological studies indicate high exposure to sunlight in early life poses an increased risk of cataract formation later in life [1]. Ultraviolet radiation with wavelengths between 290 and 320 nm (designated as UVB) is particularly relevant to cataract development [2,3], since the energy of UVB is substantially absorbed within the lens. The relevance between lens UVB absorption and cataract formation was substantially indicated in several mouse models established to elucidate genetic and biochemical factors during UVB-induced cataractogenesis [4-6].

During cataractogenesis, opacification occurs in the lens cells through degenerative mechanisms, including proteolysis, oxidation, and stress-induced protein aggregation. The detrimental effects induced by UVB irradiation act mainly through the formation of reactive oxygen species (ROS) or free radicals, such as hydrogen peroxide (H_2_O_2_), the devastatingly-toxic hydroxyl radical (OH^‧^), and superoxide (O_2_·-) [7,8]. Cataract formation, like many other degenerative ocular diseases, occurs when the rate of ROS production outpaces the rate of removal [8,9]. For example, the polyunsaturated lipids within plasma membrane of the lens fiber cells are liable to be targeted when ROS or free radicals are accumulated, leading to the initiation of early cataractogenesis. Particularly, malondialdehyde (MDA), an end product of lipid peroxidation, is increased in animal [10-13] and human cataract [14-16]. Previous studies also showed that MDA could deteriorate lens cell opacification through modification and cross-linking soluble lens proteins, such as α-crystallin or oxidative crystallins [17,18].

Without proper protection, the transparent ocular lens is doomed to be attacked by oxidative damage because the lens fiber cells are not renewed and have to last a lifetime. However, the lens is equipped with antioxidant defense mechanisms [19,20]. Under normal physiologic conditions, ROS are constitutively produced and balanced by various defense mechanisms, including antioxidant enzymes, free radical scavengers, and metal-chelating agents, that have been developed through evolution to protect cells from damage. The antioxidant enzymes include glutathione peroxidase (GPx) and superoxide dismutase (SOD). SOD catalyzes the dismutation of superoxide (O_2_-) to hydrogen peroxide (H_2_O_2_), while GPx converts H_2_O_2_ to H_2_O and O_2_ [21,22]. The free radical scavengers include ascorbate, α-tocopherol and glutathione (GSH). Particularly, GSH contains triple functions, acting not only as a free radical scavenger, but also as a regulator to regenerate other scavengers and as a substrate in the GPx reaction [21,22].

**Figure 1:**
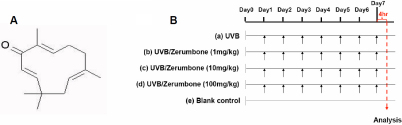
Zerumbone chemical structure and experimental design for dietary zerumbone supplementation after UVB irradiation to the mouse lens. **A**: The structure of zerumbone. **B**: Both of the mice eyes were exposed to UVB light (0.72J/cm2/daily) at noon for a consecutive 7-day period until sacrifice for analysis at 4:00 PM on Day 7.

Zerumbone (ZER; 2,6,9,9-tetramethylcycloundeca-2,6,10-trien-1-one) is a sesquiterpenoid (Figure 1A) found in large amounts in the rhizome of *Zingiber zerumbet*, a plant traditionally used in the Southeast Asian countries as an anti-inflammatory agent and also as a condiment [23-26]. Recent in vitro experiments have shown that zerumbone may be potentially used as an antitumor drug [24,27-30]. It was shown to exert anti-inflammatory effects through inhibition of nuclear factor-kappa B (NF-κB) activation and repression of the de novo synthesis of iNOS and cyclooxygenase-2 [26,27,30]. Besides, zerumbone has been widely reported to act as a free radical scavenger with antioxidant properties but without evident side effects to the normal cells [26,30-34].

Most of previous studies on zerumbone were focused on its in vitro chemopreventive and chemotherapeutic effects. The potential in vivo beneficial effects of zerumbone through its anti-oxidative characteristic have not been extensively investigated. This study investigated the effects of dietary zerumbone on UVB-induced cataractogenesis in a mouse model, since there had been no report on zerumbone application in this aspect. Our results showed that dietary zerumbone acts to inhibit opacification and reduce MDA accumulation in the eye lens following UVB exposure. Besides, we showed that dietary zerumbone can increase the production of GSH (an ROS scavenger) and GR (an anti-oxidant enzyme), following UVB exposure. Without prior publication on the same subject, these results represent the first assessment of zerumbone as a candidate agent against UVB-induced cataractogenesis.

## Methods

### Animals

A total of 50 six-week-old female ICR mice were purchased from National Laboratory Animal Center, Taipei, Taiwan. The mice weighted about 25 g on arrival and were fed ad libitum and kept under standard conditions with a 12-h light/dark cycle. The mice were acclimatized and habituated to the laboratory for at least one week before experiments. All mice were examined with a slit lamp (Model 99 BQ; Haag-Streit, Bern, Switzerland) before experiments. Only mice without anomalies of the anterior segment of the eye (cornea, anterior chamber, iris, or lens) were included in the experiments.

### Ultraviolet B irradiation and study groups

The 50 mice were randomly split into five groups (each contained 10 mice), including (1) UVB (exposure to UVB without treatment), (2) UVB/ZER (1 mg/kg; exposure to daily UVB with zerumbone treatment at 1 mg/kg bodyweight), (3) UVB/ZER (10 mg/kg; exposure to daily UVB with zerumbone treatment at 10 mg/kg bodyweight), (4) UVB/ZER (100 mg/kg; exposure to daily UVB with zerumbone treatment at 100 mg/kg bodyweight), and (5) Blank control (no daily UVB exposure and no zerumbone treatment). In the four UVB irradiation groups, the mice were anaesthetized with intraperitoneal injection of sodium pentobarbital (45 mg/kg bodyweight). Both of the mice eyes were exposed to daily UVB light from VL-6 UV lamp (set at 0.72 J/cm2; with peak wavelength at 312 nm) in the CN-6 darkroom chamber (Vilber Lourmat, Eberhardzell, Germany). Prior to UVB exposure, 0.1% tropicamide was applied to both eyes for pupil dilation. The distance between UV lamp and the mouse cornea was fixed at 16 cm. After the UVB exposure, the mice were allowed for recovery and then transferred to their original cage. The UVB irradiation was performed for a consecutive 7-day period (Day 1 to Day7 in Figure 1B). The UVB light was measured using a UV detector (VLX-3W; Vilber Lourmat) from the same company. The irradiation intensity on the cornea surface was 8mW/cm^2^ and wavelength ranged between 310 to 315 nm. All experiments were reviewed and approved by the Animal Care and Use Committee in Chung Shan Medical University and were performed in agreement with the Association for Research in Vision and Ophthalmology (ARVO) Resolution on the Use of Animals in Research.

### Dietary zerumbone supplementation

Dietary zerumbone was supplemented in mouse chow at doses of 0, 1, 10, and 100 mg per kg of bodyweight, starting from Day 0 (one day before UVB exposure) and terminated on the day of analysis (Day 7 in Figure 1B). Zerumbone was purchased from Kingherbs, Inc., Hainan, China.

### Lens opacity scoring

The extent of cataractogenesis was assessed and lens opacity was determined on day 7. Before the examination, the mice were anaesthetized with sodium pentobarbital at 30–40 mg/kg and their pupils were dilated by instillation of a 1% tropicamide and 10% phenylephrine mixture. After being anaesthetized with pupils dilated, all mice were assessed for lens opacity in left eyes. Briefly, biomicroscopy was performed in all of the five groups. The lens opacity scores were determined by using a 0 to 4 points scoring system based on the following criteria: 0=Normal, transparent lenses; 1=Lenses showing slightly hazy, translucent; 2=Lenses displaying moderate hazy, translucent; 3=Lenses displaying dense haze or point opacities; 4=Lenses displaying definite cataract [35]. The scorings were performed by 2 observers without prior knowledge of the UVB exposure and study groups. Cataract formation was photographed with a Nikon SMZ-645 dissection microscope (Nikon, Tokyo, Japan) equipped with top ring light and a digital camera.

### Determination of MDA levels

MDA levels in the lens were determined according to the procedures published by Ohkawa et al. [36], by using a fluorometric method in which 1,1,3,3 tetramethoxypropane was used as standard and the results were given as nmol/ml. Lenses were extracted from mice and homogenized in ice-cold homogenate buffer containing 0.25% sodium dodecyl sulfate (SDS), 0.65% phosphotungstic acid, 0.05 N HCL and 0.15% thiobarbituric acid (TBA). After thorough homogenization, the lens homogenate was heated and maintained at 99 °C for 45 min. After cooling, the sample was mixed with 130 μl of butanol and shaken vigorously. After centrifugation at 450× g for 10 min, the organic layer was separated and its absorbance was recorded at 555 nm. The protein levels of lens homogenate were measured with a Bio-Rad Protein Assay kit (Life Science Research, Hercules, CA) using BSA as standard, and MDA level of the lens was expressed as nmol/mg protein. Only one eye from each mouse was randomly chosen for each MDA determination to ensure independent observation.

### Quantification of GSH levels and GSH reductase activity

The lenses were extracted from mice and homogenized in ice-cold HEPES buffer (pH 7.2). The lens homogenate was then subject to centrifugation at 9,000× g for 15 min. After centrifugation, the supernatants were collected and assayed for GSH level and GSH reductase activity immediately. Tissue protein levels were measured with a Bio-Rad Protein Assay kit (Life Science Research) using BSA as standard. GSH concentration (nmole/mg) was measured according to the enzymatic recycling method of Anderson [37]. The amount of total GSH was determined based on a standard curve obtained with known amounts of GSH standards. GR activity (U/mg) was measured through the reduction of oxidized glutathione (GSSG) into GSH, which was catalyzed by GR with NADPH as the cofactor. The decrease in the optical density at 340 nm was recorded at 25 °C for 3 min and then the units of enzymatic activity were calculated using an extinction coefficient of NADPH. One unit was equivalent to the oxidation of 1 mmol of NADPH per min. Only one eye from each mouse was randomly chosen for each quantification of GSH levels or GR activity, to ensure independent observation.

### Determination of GPx and SOD activity

The lenses were extracted from mice and homogenized in ice-cold 20-mM HEPES buffer (pH 7.2) and centrifugated at 9,000× g for 15 min. After centrifugation, the supernatants were collected and assayed for GPx and SOD activity immediately. Tissue protein levels were measured with a Bio-Rad Protein Assay kit (Life Science Research) using BSA as standard. GPx activity was determined by the method of Paglia et al. [38], in which GPx was measured as a catalyst for glutathione oxidation in the presence of hydrogen peroxide (H_2_O_2_) with a spectrophotometer set at 340 nm and read against a blank reagent. GPx activity was expressed as units/mg protein (U/mg protein). One unit represents the activity of enzyme capable of converting 1 μmol of reduced glutathione (GSH) to the oxidized form of glutathione (GSSH) in the presence of H_2_O_2_/min. Superoxide dismutase (SOD) was measured according to the method of Sun et al. [39]. In this method, xanine-xantine oxidase complex produces superoxide radicals which react with nitro blue tetrazolium (NBT) to form the formazan compound. SOD activity was measured at 450 nm on a spectrophotometer by detecting the inhibition of this reaction. The activity of SOD was calculated as follows: Percent inhibition (%)=((A_blank_ – A_sample_) / A_blank_) × 100, in which A_blank_ and A_sample_ represent the absorbance values of the blank and the sample, respectively. SOD activity was expressed as units/mg protein (U/mg protein) which represents the enzyme activity capable of inhibiting 50% of the NBT reduction rate. Only one eye from each mouse was randomly chosen for each quantification of GPx or SOD activity, to ensure independent observation.

### Statistical analysis

All data were obtained from triple repeats and are presented as the means±standard error of the means (SEMs) and were compared among study groups. The lens opacity scores were compared by Mann–Whitney test. The levels of MDA, GSH, GR, GPx, and SOD were analyzed by Mann–Whitney-U test and Kruskal–Wallis test.

## Results

### Zerumbone prevents UVB-induced cataract formation as reflected by lower lens opacity scoring

**Figure 2:**
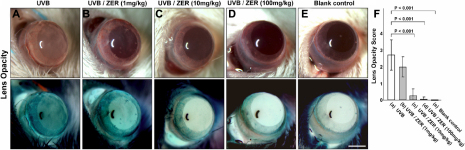
Comparison and quantification of lens opacity among groups of UVB (UVB exposure without dietary zerumbone), UVB/ZER (UVB exposure with dietary zerumbone at 1, 10, and 100 mg/kg respectively), and blank control (no UVB exposure and no dietary zerumbone). **A**-**E**: Severe cataract formation was seen in the UVB-irradiated lens in (**A**) and its corresponding negative image, which was reversed with zerumbone supplementation as shown in (**C**) and (**D**). **F**: Lens opacity scoring showed the inhibitory effect of zerumbone on cataract formation. Scale bar: 1.25 mm.

At the end of the experiment (at 4:00 PM on day 7), the mice were anesthetized with their pupils dilated for examination. The lens in UVB group showed apparent opacification (Figure 2A and its corresponding negative image), in contrast to the apparent transparent lens in the blank control group (Figure 2E and negative image). Dietary zerumbone apparently prevented the UVB-exposed lenses from cataractogenesis in a dose-dependent manner (Figure 2B-D; and negative images), with the most effective dose at 100 mg/kg. The results of lens opacity scoring were in agreement with the cataract examination (Figure 2F). Also, it appeared that dietary zerumbone started to prevent UVB-induced cataractogenesis at 10 mg/kg.

### Zerumbone reduces MDA and GSH levels in the lens with UVB irradiation

MDA level was determined to understand whether zerumbone helps to deplete its accumulation in the lens. As shown in Figure 1, MDA accumulation was significantly increased with UVB exposure, as compared to that of the blank control group (0.222±0.013 versus 0.064±0.013 nmol/mg protein; p=0.001). With dietary zerumbone supplementation, MDA levels were reduced, with significant difference starting at 10 mg/kg. (48.20% reduction), as compared to that of the UVB group without zerumbone. When dietary zerumbone was supplemented at 100 mg/kg, 76.13% reduction was achieved. In agreement with the reduction of MDA accumulation, GSH level was elevated with dietary zerumbone at 100 mg/kg when compared to that of the UVB group without zerumbone (17.71±0.99 versus 13.05±1.10 nmol/mg protein; p=0.001).

### Increase of GR and SOD and decrease of GPx activities with dietary zerumbone

Comparative to the increase of GSH, GR activity was also elevated (4.00±0.44 versus 2.02±0.51 U/mg protein; p=0.001) at 100 mg/kg of dietary zerumbone as compared to the UVB group without zerumbone. Particularly, 98.02% increase of GR activity was seen. SOD activity was also increased with 100 mg/kg zerumbone supplement as compared to that of the UVB group without zerumbone (1.32±0.21 versus 1.17±0.15 U/mg protein; p=0.115), but not as significant as the elevations of GSH level and GR activity. However, GPx activity was decreased with 100 mg/kg of zerumbone supplementation, as compared to that of the UVB group without zerumbone (3.48±0.70 versus 3.99±0.55 U/mg protein; p=0.172). Interestingly, UVB exposure leads to increase of GPx activity in the lens without zerumbone supplementation, when compared to that of the blank control (3.99±0.55 versus 3.51±0.32 U/mg protein; p=0.059).

## Discussion

Since UV exposure is inevitable, preventive measures against the phototoxic effects caused by UVB have been extensively studied. UV shields should be used for physical protection to minimize exposure. Chemically, depletion of reactive oxygen species (ROS) and free radicals and inhibition of inflammatory factors constitute two major means to ameliorate UVB-induced phototoxic status. Thus, ideally, a candidate compound should be antioxidant and anti-inflammatory. Zerumbone represents a good candidate as it contains the dual functions. Thus, this study investigated the chemical preventive effects of zerumbone against UVB-induced cataract formation. We found that zerumbone given at 100 mg/kg as a dietary supplement after UVB exposure was able to decrease the lens opacity scores (p<0.001) and to reduce MDA levels (p<0.001), in agreement with the significantly increased GSH and GR levels (both p<0.001) in the lens. Besides, SOD was also increased with dietary zerumbone at 100 mg/kg (p=0.115), but GPx (p=0.171) levels were lower as compared with those without zerumbone after UVB exposure. Out results support the potential use of zerumbone as a preventive agent against UVB-induced cataract formation.

The underlying mechanisms of zerumbone against cataract formation are likely to be derived from combinatorial effects. First, the lens, like all tissues, is equipped with endogenous antioxidant defense mechanisms to protect against the harmful effects of UVB and ROS. However, when UVB exposure becomes excessive to an extent that surpasses the endogenous defense capacity, exogenous antioxidant supplements will be determinative in cataract formation in the lens. Zerumbone has been reported to have antioxidant activity in several in vitro and in vivo cancer models [24,27,32,40-42]. Therefore, it is reasonable to interpret its preventive effects against cataractogenesis through its ability to eliminate free radicals and scavenge ROS in the lens. Second, zerumbone has been found to be able to induce de novo expression of genes encoding detoxifying/defensive proteins like phase II enzymes [32,33]. These detoxifying/defensive proteins have been known to prevent against many degenerative diseases. They are likely to act also to prevent lens degeneration and thus lens opacification was ameliorated. Third, many lines of evidence support MDA involvement in cataractogenesis. Clinically, it has been reported that products of lipid oxidation, including MDA, increase with cataract formation in the lens [14-16]. In animal studies, when oxidized phospholipids containing MDA were injected into the vitreous body of rabbits, posterior subcapsular cataract was induced within 24 h [43]. Therefore, reduction of MDA in the lens is most likely to inhibit cataractogenesis. Some researchers may doubt MDA involvement in cataractogenesis, as MDA arises only from the oxidation of polyunsaturated hydrocarbon lipids, which compose less than 3% of lens membrane lipids [44]. However, since UVB irradiation had been reported to enhance the membrane permeability of lens epithelial cells [3,45]. It is likely that, in our model, the UVB-induced MDA production in the cornea, aqueous humor, vitreous, and retina might easily diffuse to the lens, resulting in the lens opacity observed in this study. The same mechanism may also explain the risk of cataract as a consequence of retinal degeneration or retinal surgery [43,46]. Zerumbone may act to block the diffusion of MDA from the posterior ocular tissues and thus decelerate the accumulation of MDA in the lens. Alternatively, zerumbone may accelerate the depletion of MDA in the lens. These two possible mechanisms, however, remain to be elucidated by future experiments. Fourthly, cross-linking of α-crystallin by MDA has been reported previously [17,18]. Upon storage, MDA forms more reactive molecules that can cross-link proteins [17]. Therefore, MDA is not only cytotoxic to the lens cells. It could aggravate the abnormal cross-linking of soluble lens crystallins, resulting in worse lens opacification [17,18]. As such, reduction of MDA in the lens is likely to inhibit cross-linking of crystallins and therefore inhibit cataractogenesis. Fifthly, zerumbone may induce directly or indirectly the synthesis of some free radical scavengers or anti-oxidant enzymes in lens cells. Among them, GSH and GR appear to be crucial, as the indigenous levels were strongly decreased after planned UVB irradiation as compared to the blank control (both p** [a-e]=0.001; as shown in Table 1) and the decrease of GSH level and GR activity was reversed significantly by dietary zerumbone (10 and 100 mg/kg of bodyweight).

**Table 1:** Antioxidnat concentration (level) in lens.

** **	**MDA (nmol/mg protein)**	**GSH (nmol/mg protein)**	**GR (U/mg protein)**	**SOD (U/mg protein)**	**GPx (U/mg protein)**
(a) UVB	0.22±0.01	13.05±1.10	2.02±0.51	1.17±0.15	3.99±0.55
(b) UVB/Zerumbone (1mg/kg)	0.19±0.03	12.95±1.56	2.77±0.67	1.25±0.15	3.76±0.49
(c) UVB/Zerumbone (10mg/kg)	0.12±0.01	15.35±1.03	3.16±0.41	1.27±0.13	3.55±0.48
(d) UVB/Zerumbone (100mg/kg)	0.05±0.01	17.71±0.99	4.00±0.44	1.32±0.21	3.48±0.70
(e) Blank control	0.06±0.01	18.15±1.07	4.03±0.48	1.37±0.15	3.51±0.32
**Percent change**					
(a-b)	-12.61	-0.77	37.13	6.84	-5.76
(a-c)	-48.20	17.62	56.44	8.55	-11.03
(a-d)	-76.13	35.71	98.02	12.82	-12.78
p* (a-b-c-d-e)	0.000	0.000	0.000	0.154	0.303
p** (a-b)	0.248	0.834	0.115	0.172	0.345
p** (a-c)	0.001	0.003	0.001	0.172	0.074
p** (a-d)	0.001	0.001	0.001	0.115	0.172
p** (a-e)	0.001	0.001	0.001	0.016	0.059

p*; Kruskal Wallis test; p**; Mann Whitney U test.

Adequate nutrition is considered particularly important in preventing ROS-induced oxidative damage and maintaining the overall health of the eye [47-49]. Many of the enzymatic cofactors and chemical constituents necessary for antioxidant activity are obtained through the diet. For example, dietary treatment with melatonin was reported to reduce UVB-induced cataract formation in the experimental animal studies [50-53]. Our study has shown, for the first time, that dietary zerumbone can dramatically inhibit lens opacity after UVB irradiation. Particularly, previous studies had indicated that zerumbone did not cause any harmful effects in the liver of normal rats [54]. Notably, we fed the mice with zerumbone for only 7 days with a maximal dose at 100 mg/kg. Taha et al. [54] performed intraperitoneal zerumbone treatment at 60 mg/kg twice a week for 11 weeks and found no evidence of abnormality in the liver of normal rats. Another study by Mihye Kim et al. [29] used dietary zerumbone at 500 ppm for 17 weeks without finding inflammatory response, mucosal ulcer, or high grade dysplasia in the digestive system. It is likely that the preventive dose of dietary zerumbone against ultraviolet B-induced cataractogenesis may not cause toxic effects, although caution must certainly be taken.

We conclude that zerumbone may be potentially used as a food supplement to prevent against UVB-induced cataract formation.
